# Flora Capture: a citizen science application for collecting structured plant observations

**DOI:** 10.1186/s12859-020-03920-9

**Published:** 2020-12-14

**Authors:** David Boho, Michael Rzanny, Jana Wäldchen, Fabian Nitsche, Alice Deggelmann, Hans Christian Wittich, Marco Seeland, Patrick Mäder

**Affiliations:** 1 Institute for Computer and Systems Engineering, Technische Universität Ilmenau, Helmholtzplatz 5, 98693 Ilmenau, Germany; 2 Department Biogeochemical Integration, Max-Planck-Institute for Biogeochemistry, Hans-Knöll-Str. 10, 07745 Jena, Germany

**Keywords:** Structured plant observations, Multi-organ plant identification, Mobile app, Citizen science, Digital plant collection, Digital herbariumn

## Abstract

**Background:**

Digital plant images are becoming increasingly important. First, given a large number of images deep learning algorithms can be trained to automatically identify plants. Second, structured image-based observations provide information about plant morphological characteristics. Finally in the course of digitalization, digital plant collections receive more and more interest in schools and universities.

**Results:**

We developed a freely available mobile application called Flora Capture allowing users to collect series of plant images from predefined perspectives. These images, together with accompanying metadata, are transferred to a central project server where each observation is reviewed and validated by a team of botanical experts. Currently, more than 4800 plant species, naturally occurring in the Central European region, are covered by the application. More than 200,000 images, depicting more than 1700 plant species, have been collected by thousands of users since the initial app release in 2016.

**Conclusion:**

Flora Capture allows experts, laymen and citizen scientists to collect a digital herbarium and share structured multi-modal observations of plants. Collected images contribute, e.g., to the training of plant identification algorithms, but also suit educational purposes. Additionally, presence records collected with each observation allow contribute to verifiable records of plant occurrences across the world.

## Background

**Fig. 1:**
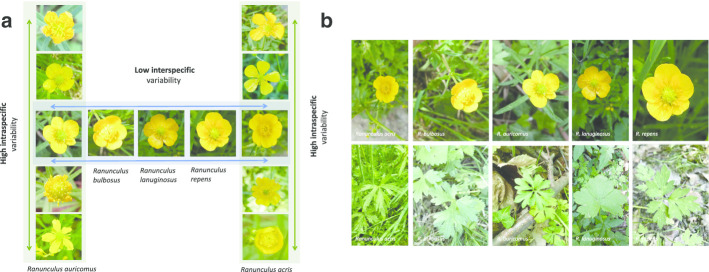
**a** Interspecific variability across five *Ranunculus* species and intraspecific variability within two of the species. **b** Five *Ranunculus* species hardly distinguishable by their flowers alone, but clearly identifiable when accompanied by leave images

Efforts to automatically identify species from images have substantially increased in recent years [1, 2]. Deep learning methods revolutionize our ability to train computers in identifying organisms from image data, such as insects [3], fishes [4], plankton [5], mammals [6] and plants [7]. Specifically, convolutional neural networks (CNNs) allow for superior recognition performance [8, 9] and form the basis for successful automated plant species identification [1, 10]. Deep CNNs have been demonstrated to facilitate classification accuracies that are on par with human performance for general object recognition tasks [8] as well as for fine-grained species identification tasks [11]. Latest studies on automated image-based plant identification show identification accuracy that at least reaches humans’ identification abilities for common plants [1, 7, 12]. However, with more than 380,000 described species worldwide [13], automated plant identification still constitutes a challenging image recognition problem, further complicated by low interspecific variability and high intraspecific variability for many species (cp. Fig. 1).

CNNs rely on vast numbers of training images. While the algorithms themselves are constantly refined and improved [1, 14], the accuracy of identification is strongly dependent on the quality of images used in the training as well as the eventual identification process. Therefore, distinguishing very similar plants requires the provision of suitable training images depicting species-specific details. Often, a single image is not enough to reliably identify a species (cp. Fig. 1). This is true for humans as well as for algorithms [15] and accuracy of species identification has been shown to be considerably improved by analyzing more than one image perspective in an identification process [7, 16, 17]. However, most plant image datasets (e.g., GBIF [18], iNaturalist [19], Pl@ntNet [20]) only contain one image per observed plant and were not collected in a structured manner. While prominent organs such as the flower of angiosperms are well populated, other organs such as leaves and fruits are often underrepresented or even missing. Furthermore, the majority of images belongs to single-image observations which, in many cases, do not allow for a safe identification, especially if leaves and other important details are not depicted [15]. Unfortunately, the frequency of misidentifications is proportional to the difficulty of discriminating species. Therefore, the ability of algorithms to distinguish visually similar species is further reduced through the presence of incorrectly labelled training data. Structured multi-image observations of plants, following predefined perspectives, provide solid classification results even for species showing strong visual resemblance, such as grasses [7]. Within a structured observation also images depicting less prominent perspectives can safely be identified and labeled, which might not be possible without their accompanying perspectives. Having these perspectives as training data, eventually enables plant identification algorithms to identify plants based on less conspicuous vegetative parts. The creation of structured image data sets is an important task to further improve automatic plant recognition.

Upcoming trends in crowdsourcing and citizen science offer excellent opportunities to generate and continuously update image datasets. Advances in mobile technology and the ubiquity of smartphones provides billions of potential users with a powerful tool to record, collect and share images of species surrounding them. Members of the public are therefore able to acquiring data and to contribute to scientific research projects with very little required knowledge of the subject. Involving citizen to accumulate such structured observations can produce a large and high-quality dataset [21]. Keeping participants engaged is challenging but can be accomplished by providing constant feedback of their performance [22]. In the following section, we introduce the mobile application Flora Capture, allowing users to create structured plant observations and to submit them to the servers of the Flora Incognita research project. Depending on a plant’s life form, Flora Capture guides a user through a sequence of suitable plant perspectives to be captured. These perspectives have been carefully selected and evaluated in order to capture complementary information making up an observation likely containing sufficient information for an expert botanist to identify or verify a provided id of the depicted plant [7, 23]. Currently, each captured and accepted observation contributes to a dataset used for improving the Flora Incognita plant identification app [24]. Additionally, Flora Capture has been used to conduct other studies and may be used in various ways to support new studies [7].

## Implementation

### Architectural overview

**Fig. 2:**
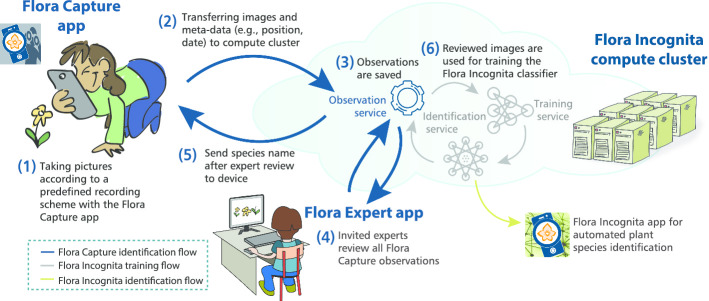
Architectural overview of the Flora Capture system

We designed the Flora Capture system as a flexible client–server solution consisting of scalable micro services running in our data center and client applications realizing different plant identification scenarios (see Fig. 2). Below, we briefly describe the system’s modules.

*Flora Capture app* The Flora Capture app is a multi-platform app, freely available on Android and iOS with a modular code base ensuring maximum reuse and consistency of functionality across the different applications developed within the project. Flora Capture enables users to take offline multi-image observations that are identified batch-wise upon sync to our server and allow for creating a digital plant collection.

*Observation service* After synchronization, observations collected with Flora Capture are handed over to an observation service. An observation consists of several images from predefined perspectives as well as provenance data (device, author, date, etc.). Each observation is optionally associated with a geolocation and a species name. Each observation not identified by the user themselves will instantly be analyzed by an automated identification service providing an initial feedback to the user upon synchronization. This feedback is conservative meaning that the result is only reported if the classifier is very confident in its decision. In addition to this initial feedback, each uploaded observation will be reviewed by botanical experts using our Flora Expert app. Each confirmed observation can then be used for further analysis. Currently, we use them as training images for the Flora Incognita identification service [24].

**Fig. 3:**
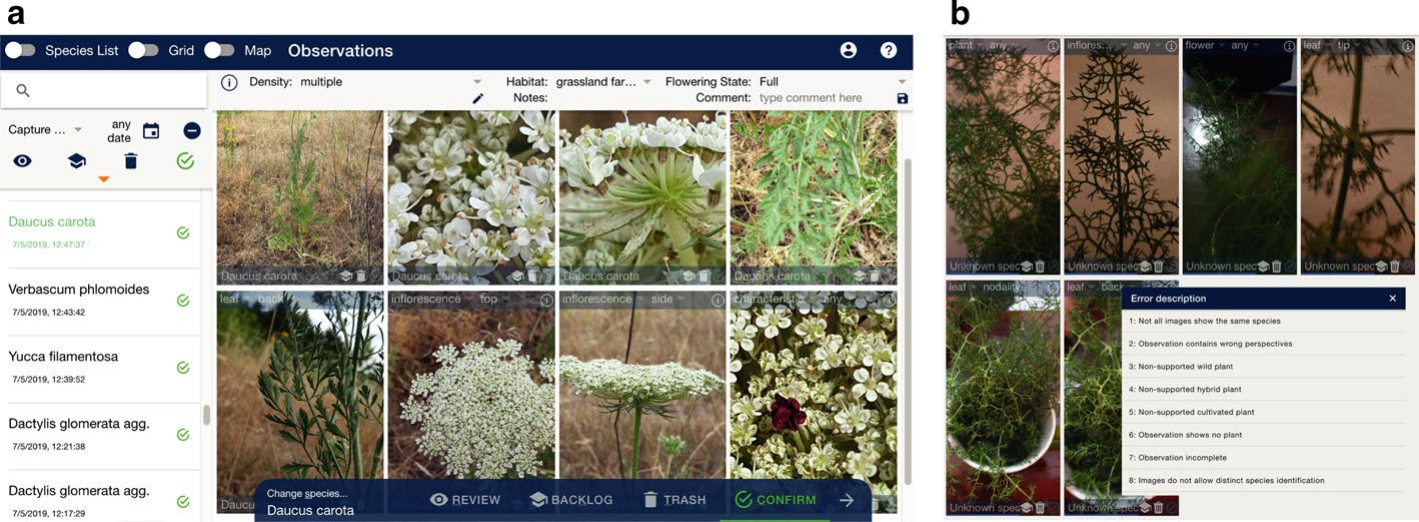
**a** Main Flora Expert user interface showing a *Daucus carota* observation. **b** Experts rejecting incorrect observations can select from a list of predefined problems and can also add individual feedback to guide the user

*Flora Expert app* Flora Capture relies on image reviews and eventual species identifications that are solely conducted by botanical experts associated with the project. We are continuously expanding this group of expert reviewers but plan to only involve specialists. We argue that involving an open community may introduce too much noise and potentially wrong labels into the acquired data. For the purpose of reviewing incoming Flora Capture observations, we have developed the Flora Expert app available as website and on mobile devices. Using the app, our experts can confirm, relabel, postpone and reject observations on a per image level (cp. Fig. 3). Observations that cannot be identified by our experts, either because an exact species cannot unambiguously be determined or the observation’s quality is not suitable, will be rejected (cp. Fig. 3b). We performed a cross-evaluation, where each of our experts re-reviewed 50 randomly drawn observations without knowing the result of a previous review conducted by another expert. These independent results matched in 96% of the evaluated observations. The differences were mainly due to fact that different experts have different experiences for particular plant groups. In reality, critical observations would be discussed among different experts to reach a consensus decision.

In order to engage and teach our Flora Capture users, feedback on their observations including the final species label is transferred back to their device. The *My Observations* list within the app shows all observations collected by a user and a color-coded symbol indicates their review status, i.e., not-synced, under review, accepted, rejected. In addition, the user receives a lot of information about the species, e.g. protection status, characteristics, distribution maps. If our experts reject an observation, the user that contributed it will see detailed information why her or his observation did not meet the project’s general acceptance criteria and, if required, an individual message (cp. Fig. 3b).

### How Flora Capture works

**Fig. 4:**
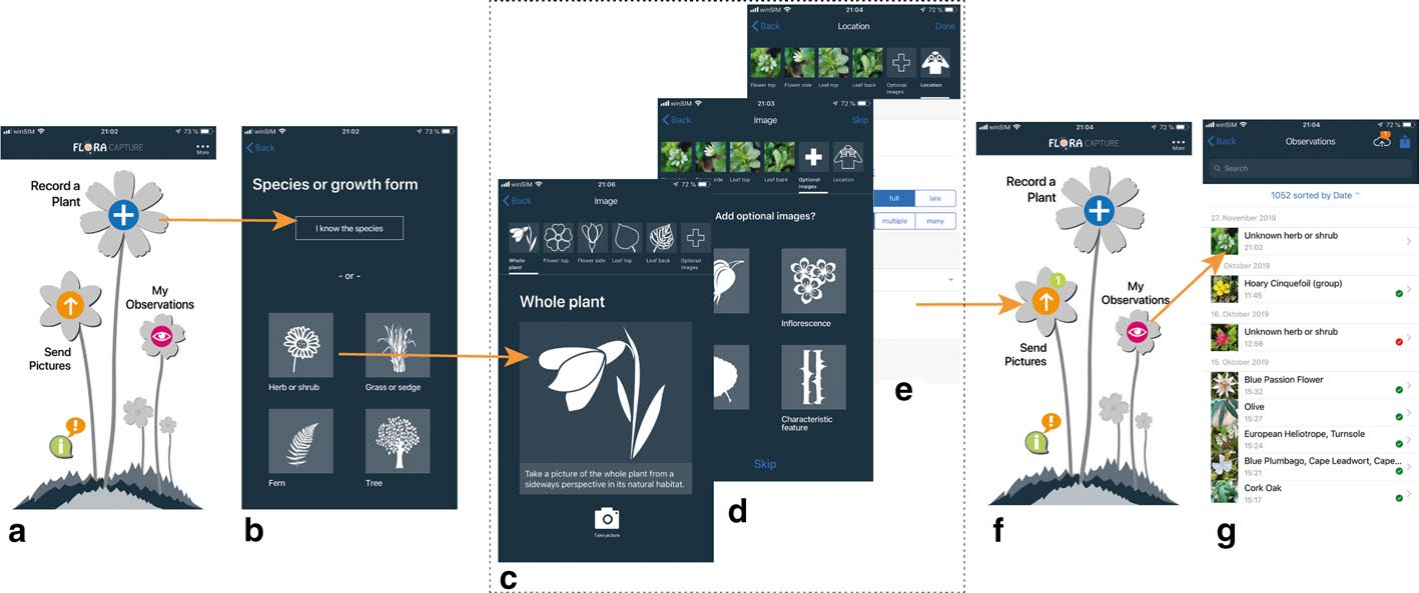
Taking an observation with Flora Capture illustrated for an unknown species with growth form “Herb or shrub”

After downloading the app and registering, users can create new observations, sync existing observations and view all previously taken observations (cp. Fig. 4a). When creating a new observation a user will be guided step-by-step in a questionnaire-like manner suitable for laymen as well as for experts. If a user is able to identify the plant to be observed or has a hypothesis about its species, she or he may provide it—if not, she or he may choose to only enter its growth form (cp. Fig. 4b, Table 1). Depending on the growth form, either directly entered by the user or derived form the selected species, we ask a user to take images of the plant to be observed from the most suitable perspectives. In order to reduce users’ cognitive load when using the app, we group questions logically and continuously inform about the overall progress visually. Figure 4 shows an example workflow for the *herb or shrub* growth form. Each growth form is associated with a number of mandatory perspectives and may additionally offer optional perspectives that a user may or may not acquire (cp. Table 1). For example, an additional second leaf could be recorded if a plant has differently shaped basal and stem leaves. An optional perspective called *characteristic feature* allows users to capture specifics of a plant that she or he deems particularly relevant for identification of the species and that have not been captured sufficiently in any of the other perspectives (cp. Fig. 4d). After taking the mandatory and potentially further optional images, we display a short questionnaire inquiring a rough estimate of the number of individuals of the same species on the site, the flowering state of the observed plant, and a classification of the habitat (cp. Fig. 4e). Additionally, users may take individual notes. Filling this questionnaire completes an observation. A complete observation consists of all images, the information acquired via the questionnaire and metadata, such as time, date, and geolocation of the observation. With this final step, the user lands back on the main screen of the app, showing the number of pending observations to be synchronized to the server (cp. Fig. 4f). Decoupling recording and synchronization of observations enables users to take observations at remote locations without network coverage and to synchronize them once they are back home and have a stable internet connection.

Users can delete their observations before transferring them to the server. In this case, the observation and all its content will be deleted entirely and never synced. Once transferred to the server, users can still remove observations from their profile. Such observation will not further be shown on a user’s devices, not reviewed and not used for further analyses. Since any observation is expert-reviewed before further usage, we do strictly remove any image that shows humans or other non-plant objects, except hands holding a plant. Users find a consistent privacy statement across the app, the app stores and the project’s webpage explaining which information is being collected by the app and how it may be used. We explain that the collected data is used for research purposes, that we share observation data with conservation authorities and that collected observation data may in an anonymized form be released as datasets at a later point in time.

**Table 1:** Observation recording scheme for different plant growth forms

Perspective	Growth form			
	Herb or shrub	Tree	Grass or sedge	Fern
Mandatory	Whole plant	Stem	Whole grass	Whole fern
	Flower top	Bark	Inflorescence	Leaf tip
	Flower side	Leaf top	Flower	Leaf back
	Leaf top	Leaf back	Leaf tip	
	Leaf back		Ligule	
			Leaf back	
Optional	Fruit	Whole tree		
	Inflorescence top	Fruit		
	Inflorescence side	Bud		
	Additional leaf top			
	Additional leaf back			
	Characteristic feature			

### Using Flora Capture to acquire plant observations

There are multiple ways in which Flora Capture can be used to acquire plant observations for a new research purpose. First, users can export all as well as filtered subsets of their observations from all their devices as a comma-separated values (CSV) file containing all meta-data (i.e., species id, date, location) and can also export the associated images. Second, groups of users may share one Flora Capture account in order to independently collect plant observations with their mobile devices, have them reviewed by our experts, share them among another and eventually export observation data as described before. Third, we are open for scientific collaborations by setting up new observation projects and based on already collected observation data. For example, we recently established a collaboration with an agricultural school in Austria where students create digital plant collections replacing the traditional task of excavating and collecting plant specimen in order to prepare herbarium sheets. In 2020, the most motivated students collected up to 200 plant observations being much more than required to fulfill the school’s task. Another example is a recent study that used Flora Capture observations to explore the image perspectives’ information content for automated plant identification [7]. This experiment follow a strict protocol asking study participants to collect observations of 100 species explicitly containing such that are easily confused, i.e., many congeneric species and 12 Poaceae. In total, 10,000 observation have been collected. This study found, e.g., that a combination of the front and lateral perspectives of flowers and the top perspective of leaves allow accuracies greater than 95%.

**Fig. 5:**
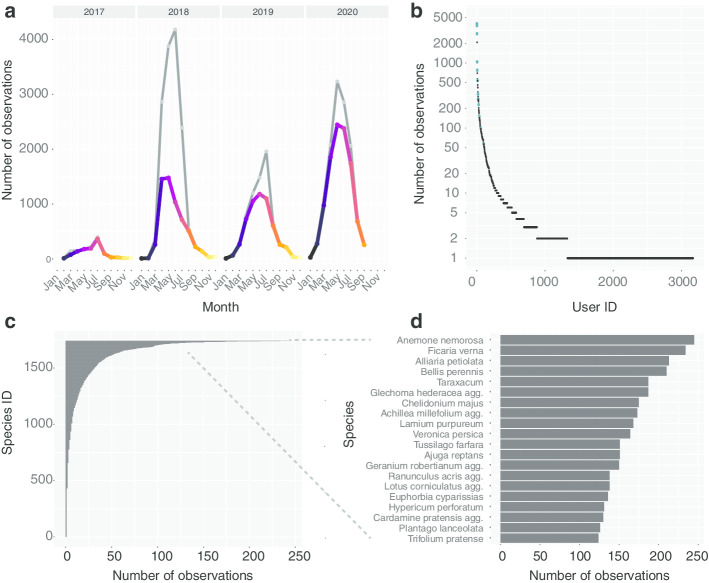
**a** Flora Capture observations contributed by citizen scientists (colored line) and by all users including project members and partners (grey line) per month since January 2017. **b** Number of valid observations collected per user ID. Blue points belong to IDs associated with project members. **c** Number of observations per species collected so far by citizen scientists. **d** Top 20 most frequently observed species by Flora Capture citizen scientist users

Flora Capture additionally offers a blog called *stories* in which we provide general tips for improving the quality of observations but also guide citizen in collecting specific species. In a story series called “Species of the month”, we present species where more information about their distribution and more images depicting their trait expressions are desired. Indeed, some of the advertised “Species of the month” do occur now in the most observed species (e.g.: *Anemone nemorosa, Ficaria verna, Alliaria petiolata, Glechoma hederacea* and others; Fig. 5d). Demonstrating that the blog allows communicating current research goals to the citizen. We consider providing a clear focus important for keeping users involved in the research process. Stories are an ideal measure to communicate new research objects and to encourage existing app user to collect observations supporting this research.

## Results

Since the release of Flora Capture in October 2016, we collected more than 40,000 accepted plant observations. As of September 2020, these comprise more than 200,000 images and cover more than 1890 plant species (cp. Fig. 5). The number of external contributions by citizen scientists amounts to more than 5000 observations per year (2017: 1158; 2018: 5919; 2019: 5542; 2020 (as of September): 10,640). More than 3000 distinct user IDs contributed at least one valid plant observation until now and about 500 users uploaded more than 5 observations (eg.: Fig. 5b). The most active citizen scientists have contributed hundreds (in one case even more than 2000) of high quality observations since app release. The distribution of observed plants is highly skewed (Fig. 5c), where the most frequently observed species are common, broadly distributed and conspicuous species. The majority of of observed species represent ruderal and nitrophilic species often found on roadsides and farmland (Fig. 5d). A majority of observations originates from within Germany where the app is being developed. Here, members and partners of the Flora Incognita project are active in promoting the app and getting in touch with potentially interested citizen scientists. However, an increasing number of observations is also taken in other parts of Europe and across the world. Flora Capture is currently available in eleven different languages and the number of observed species is steadily increasing.

## Discussion

Active facilitation of research projects involving citizen scientists can ultimately inspire individual behavior and encourage public action with respect to conservation efforts [25]. The Flora Capture app provides a convenient tool for users to rise their awareness for plant diversity in their surroundings and buildup knowledge about plants. Users that take images of a plant from different perspectives, eventually touch the leaves to arrange them appropriately or look for specific characteristics inevitably sharpen their eye for details characterizing the plant of interest. At the same time, Flora Capture also allows to increase the quality of automated species identification by creating a high quality image dataset [7]. Training images for CNNs are required to be highly variable as well as informative in order to provide reliable results when distinguishing very similar species. Images generated by thousands of users inherently increase the variability of data through the diversity of the contributing users [26]. The diversity and quality of image contributors will presumingly match the quality and diversity of the images which are to be identified. At the same time, the structured approach of acquiring predefined perspectives combined with an expert review ensures high reliability and information-richness in the acquired image data [27]. The metadata collected with each observation additionally provides valuable information supporting the identification process with new aspects to consider (e.g. location, time of the year). In conclusion, the proposed multi-modal approach for recording plant observations allows creating a verifiable plant observation database and serves as an important source of information on its own [28, 29].

### Future directions

Currently, Flora Capture supports 4800 species mainly distributed in central Europe. Future versions of the app will successively cover a larger pool of species and potentially require a revision of the available growth forms. Furthermore, the observation protocol will optionally be extended with a standardized mapping protocol, allowing plant mappers to provide even more detailed descriptions of their observations and allow the use of Flora Capture in vegetation mapping campaigns. In fact, structured image databases for other kinds of organisms can be developed in a similar way. Physical species records in biological collections could be supplemented with in-situ observation data [30]. Current pilot projects in schools and universities where students are using Flora Capture on botanical excursions show encouraging results. Students learned to take close looks at plants while taking pictures for their digital observations. In subsequent presentations and discussions with classmates, it became clear that fusing botany and digital tools was a reliable approach to spark interest in plant taxonomy. Last but not least, various passionate users have reported that spending time in nature and collecting plant observations using Flora Capture is beneficial for their health and inspires learning. Identifying plants encourages users to collect even more observations. In fact, smartphone applications offer a great potential to engage users via gamification [31, 32]. Providing convincing, appealing and well implemented applications involving plants instead of Pokémons in nature might even inspire previously uninvolved citizen scientists to participate.

## Availability and Requirements

- Project name: Flora Capture
- Project home page: https://www.floraincognita.com/flora-capture-app/
- Operating systems: Android 4.4W or higher, iOS 10 or higher
- Programming languages: TypeScript, Swift, Kotlin, SQL, PHP, Python
- License: https://www.apple.com/legal/internet-services/itunes/dev/stdeula/ Any restrictions to use by non-academics: no

## Data Availability

App usage data analyzed within this paper are available from the corresponding author on reasonable request.

## Abbreviations

CNN: Convolutional neural network
CSV: Comma-separated values
